# Supplementary material for: Effect of frequency and rhythmicity on flicker light-induced hallucinatory phenomena
Source: PLoS One. 2023 Apr 11;18(4):e0284271. doi: 10.1371/journal.pone.0284271 (PMC10089352; doi:10.1371/journal.pone.0284271)
Supplement: S1 Table — (PDF) [file pone.0284271.s001.pdf]

S2 Tables

Table 1: Tukey tests of SVES Simple Visual Hallucination ratings

Factor 1	*Factor 2 (I)*	*Factor 2 (J)*	*Mean difference (I-J)*	*CI Lower bound*	*CI Upper bound*	*P value*
3 Hz	Rhythmic	$\text{Arrhythmic}_{pairs}$	0.65	-16.33	17.62	1.0
		Arrhythmic_{norm}	1.22	-15.75	18.20	1.0
8 Hz		$\text{Arrhythmic}_{pairs}$	19.14	2.17	36.12	0.01
		Arrhythmic_{norm}	6.89	-10.08	23.87	1.0
10 Hz		$\text{Arrhythmic}_{pairs}$	34.56	17.58	51.54	<0.001
		Arrhythmic_{norm}	15.98	-0.10	32.96	0.09
18 Hz		$\text{Arrhythmic}_{pairs}$	21.07	4.09	38.04	0.003
		Arrhythmic_{norm}	12.33	-4.64	29.31	0.4
Rhythmic	18 Hz	10 Hz	2.57	14.41	19.54	1.0
		8 Hz	12.05	4.92	29.03	0.4
		3 Hz	42.15	25.17	59.12	<0.001
	10 Hz	8 Hz	14.62	2.35	31.60	0.2
		3 Hz	44.71	27.73	61.69	<0.001
	8 Hz	3 Hz	30.09	13.12	47.07	<0.001

CI, 95% Confidence interval.

Table 2: Tukey tests of SVES Dynamics ratings.

Factor 1	*Factor 2 (I)*	*Factor 2 (J)*	*Mean difference (I-J)*	*CI Lower bound*	*CI Upper bound*	*P value*
3 Hz	Rhythmic	$Arrhythmic_{pairs}$	3.31	-19.21	25.84	1.0
		$Arrhythmic_{norm}$	4.23	-18.31	26.76	1.0
8 Hz		$Arrhythmic_{pairs}$	31.10	8.57	53.63	<0.001
		$Arrhythmic_{norm}$	14.68	-7.86	37.21	0.6
10 Hz		$Arrhythmic_{pairs}$	46.19	23.66	68.72	<0.001
		$Arrhythmic_{norm}$	23.48	0.94	46.01	0.03
18 Hz		$Arrhythmic_{pairs}$	31.49	8.96	54.02	<0.001
		$Arrhythmic_{norm}$	18.86	-3.67	41.39	0.2
Rhythmic	18 Hz	10 Hz	-4.49	-27.02	18.04	1.0
		8 Hz	13.05	-9.48	35.58	0.8
		3 Hz	55.31	32.78	77.84	<0.001
	10 Hz	8 Hz	17.54	-4.99	40.07	0.3
		3 Hz	59.80	37.27	82.33	<0.001
	8 Hz	3 Hz	42.26	19.73	64.79	<0.001

CI, 95% Confidence interval.

Table 3: Tukey tests of SVES Detail ratings

Factor 1	*Factor 2 (I)*	*Factor 2 (J)*	*Mean difference (I-J)*	*CI Lower bound*	*CI Upper bound*	*P value*
3 Hz	Rhythmic	$Arrhythmic_{pairs}$	6.73	-18.97	32.42	1.0
		$Arrhythmic_{norm}$	3.30	-22.39	28.99	1.0
8 Hz		$Arrhythmic_{pairs}$	19.38	-6.32	45.07	0.4
		$Arrhythmic_{norm}$	6.95	-18.74	32.64	1.0
10 Hz		$Arrhythmic_{pairs}$	22.78	-2.92	48.47	0.1
		$Arrhythmic_{norm}$	13.30	-12.39	38.99	0.9
18 Hz		$Arrhythmic_{pairs}$	18.05	-7.64	43.74	0.5
		$Arrhythmic_{norm}$	9.78	-15.92	35.47	1.0
Rhythmic	18 Hz	10 Hz	1.18	-24.52	26.87	1.0
		8 Hz	13.33	-12.37	39.02	0.9
		3 Hz	42.48	16.78	68.17	<0.001
	10 Hz	8 Hz	12.15	-13.54	37.84	0.9
		3 Hz	41.30	15.61	66.99	<0.001
	8 Hz	3 Hz	28.88	3.37	54.38	0.01

CI, 95% Confidence interval.

Table 4: Tukey tests of SVES *Kluver form* ratings

Factor 1	*Factor 2 (I)*	*Factor 2 (J)*	*Mean difference (I-J)*	*CI Lower bound*	*CI Upper bound*	*P value*
3 Hz	Rhythmic	$\text{Arrhythmic}_{pairs}$	-1.72	-21.70	18.26	1.0
		Arrhythmic_{norm}	0.11	-19.87	20.09	1.0
8 Hz		$\text{Arrhythmic}_{pairs}$	16.72	-3.26	36.70	0.2
		Arrhythmic_{norm}	4.98	-15.00	24.96	1.0
10 Hz		$\text{Arrhythmic}_{pairs}$	34.82	14.84	54.80	<0.001
		Arrhythmic_{norm}	14.47	-5.51	34.45	0.4
18 Hz		$\text{Arrhythmic}_{pairs}$	21.59	1.61	41.57	0.02
		Arrhythmic_{norm}	12.75	-7.23	32.73	0.6
Rhythmic	18 Hz	10 Hz	-1.04	-21.02	18.93	1.0
		8 Hz	15.08	-4.88	35.07	0.3
		3 Hz	44.69	24.72	64.67	<0.001
	10 Hz	8 Hz	16.08	-3.70	35.86	0.2
		3 Hz	45.32	25.54	65.10	<0.001
	8 Hz	3 Hz	29.60	9.62	49.58	<0.001

CI, 95% Confidence interval.

Table 5: Tukey tests of SVES *Other form* ratings

Factor 1	*Factor 2 (I)*	*Factor 2 (J)*	*Mean difference (I-J)*	*CI Lower bound*	*CI Upper bound*	*P value*
3 Hz	Rhythmic	$Arrhythmic_{pairs}$	3.75	-16.14	23.87	1.0
		$Arrhythmic_{norm}$	0.70	-19.42	20.82	1.0
8 Hz		$Arrhythmic_{pairs}$	17.95	-2.17	38.07	0.1
		$Arrhythmic_{norm}$	8.23	-11.90	28.34	1.0
10 Hz		$Arrhythmic_{pairs}$	31.20	11.08	51.32	<0.001
		$Arrhythmic_{norm}$	13.40	-6.72	33.52	0.6
18 Hz		$Arrhythmic_{pairs}$	17.01	-2.89	36.90	0.2
		$Arrhythmic_{norm}$	7.09	-12.80	26.98	1.0
Rhythmic	18 Hz	10 Hz	-3.89	-24.01	16.24	1.0
		8 Hz	9.41	10.71	29.54	0.1
		3 Hz	34.24	14.11	54.36	<0.001
	10 Hz	8 Hz	13.30	-6.82	33.42	0.6
		3 Hz	38.13	18.00	58.25	<0.001
	8 Hz	3 Hz	24.49	4.59	44.38	0.003

CI, 95% Confidence interval.

Table 6: Tukey tests of SVES *Complex Imagery ratings*

Factor 1 (I)	*Factor 1 (J)*	*Mean difference (I-J)*	*CI Lower bound*	*CI Upper bound*	*P value*
18 Hz	10 Hz	2.73	-14.75	20.20	1.0
	8 Hz	4.45	-13.02	21.92	1.0
	3 Hz	15.98	-1.50	33.45	0.1
10 Hz	8 Hz	1.73	-15.75	19.20	1.0
	3 Hz	13.25	-4.22	30.72	0.3
8 Hz	3 Hz	11.53	-5.95	29.00	0.6

CI, 95% Confidence interval.
