# Supplementary material for: Effect of frequency and rhythmicity on flicker light-induced hallucinatory phenomena
Source: PLoS One. 2023 Apr 11;18(4):e0284271. doi: 10.1371/journal.pone.0284271 (PMC10089352; doi:10.1371/journal.pone.0284271)
Supplement: S1 Appendix — (PDF) [file pone.0284271.s002.pdf]

<u>Stroboscopic Visual Experience Scale (SVES) – Abridged version</u>

Instruction

This survey will ask you about your experience during the stimulus period.
The first page will ask about the visual elements of your experience. What you saw will be referred to as "your visual experience".
Questions may be illustrated with images, usually consisting of dotted lines. For example, you may be asked if you saw circles with an image like this:

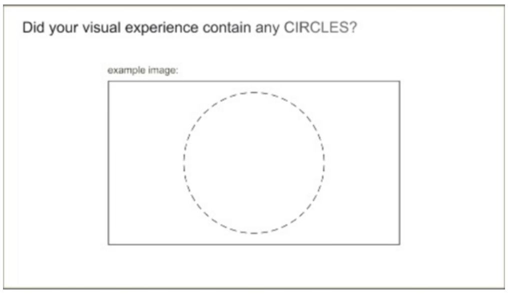

These images are purely illustrative. You should always answer to the meaning of the question. You should ignore aspects of the example images that are not relevant to the question, such as the nature of the dotted lines.
if you saw any of these, you should answer yes, you saw circles:

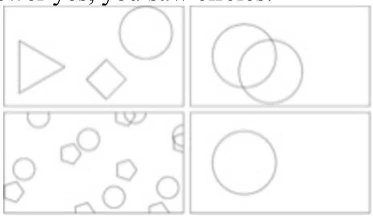

if you saw these, you should answer no, you did not see circles:

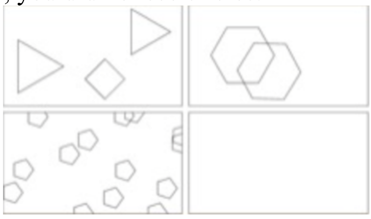

You will also be asked questions where the illustrations span a spectrum. For example:

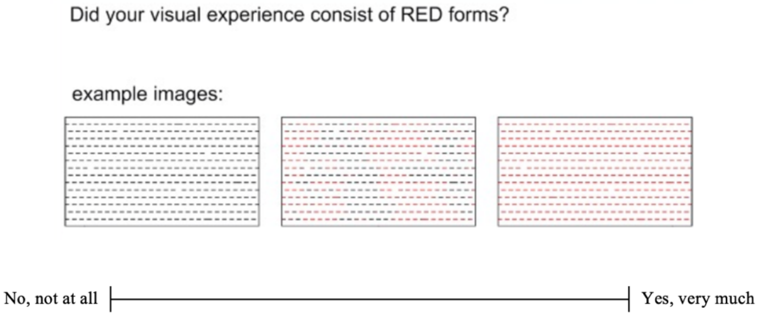

As always, you should answer based on the meaning of the text, using the illustrations only as a rough guide.
Here, for example, we are interested in whether you saw redness, rather than dashed lines.
The "yes, very much so" end of the spectrum consists of images that answer the question perfectly.

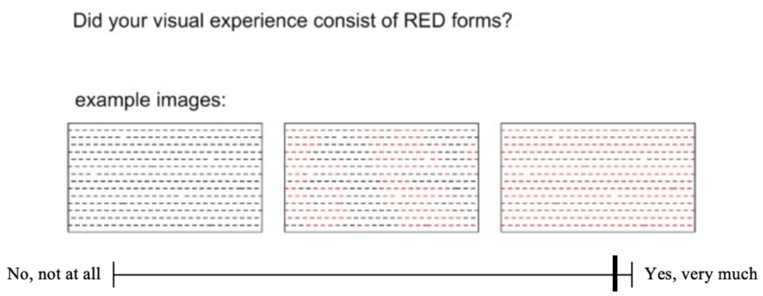

In this case, this consists of images made up of red forms, shapes, lines etc. Example "yes" images:

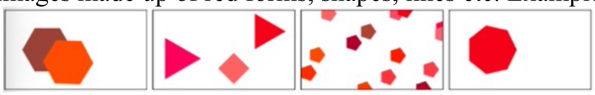

The "no, not at all" end of the spectrum can consist of anything that doesn't fit within the text of the question. It doesn't need to look anything like the example image.

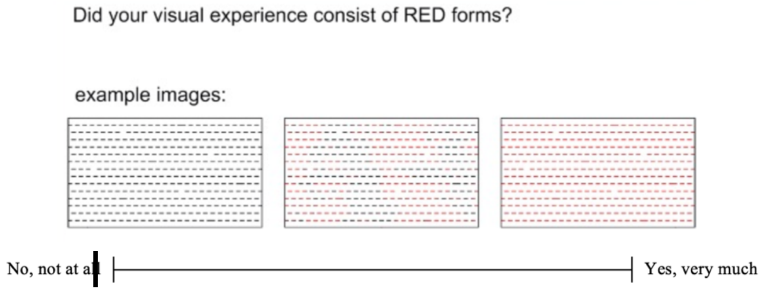

In this case, that means images without red, including any other colours besides red, or nothing at all. Example "no" images:

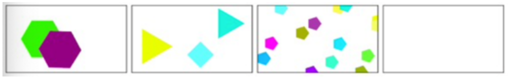

The middle areas of the spectrum can consist of anything in between.

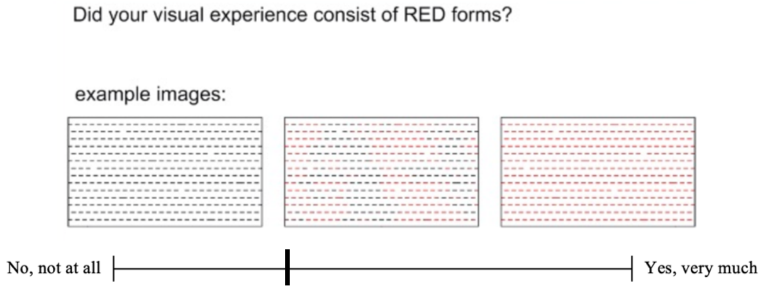

In this case, that means images with red forms and non-red forms (any other colour), such as:

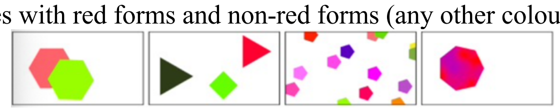

Within this implementation of the survey, the spectrum will range from 0%, which represents "no, not at all", and 100%, corresponding to "yes, very much". You will be able to give a percentage answer anywhere between 0 and 100, where answers below 50% tend towards "no" and answers above 50% tend towards "yes". Initially, there will be no wedge present on the slider. It will look like this:

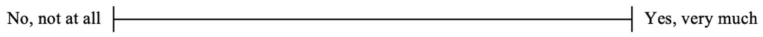

However, once you click anywhere along the slider, the wedge will appear and you will then be able to move it up and down the slider to choose your final answer. Here is an example:

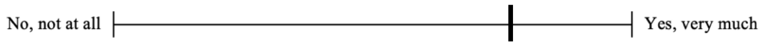

The subsequent page will present a series of statements about your experience. You should answer based on whether you agree or disagree with the statement on the spectrum from "no, not more than usually", which will also be represented as 0%, to "yes, much more than usually", which is given as 100%. As above, the slider will allow any integer between 0 and 100. The wedge will begin at 0, representing an experience that did not deviate from normal waking consciousness. Here is an example:

Example: I could see images from my memory or imagination with extreme clarity

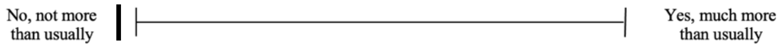

Visual Experience Scale - Items

Item 1: How well do you recall your visual experience right now?

Not at all ⊢——————————⊣ Perfectly

Item 2: What colours did you see? Select all that apply:

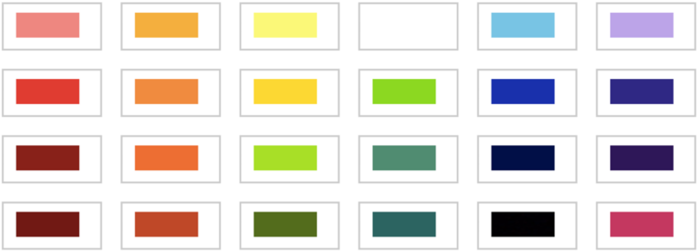

Item 3: Did you see any grids or forms aligned to a grid?

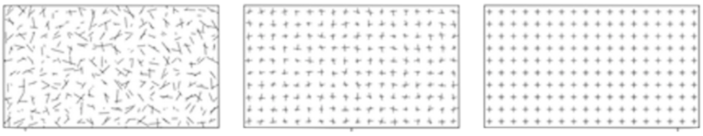

No, not at all ⊢——————————⊣ Yes, very much

Item 4: Did you see any formations like targets or cobwebs?

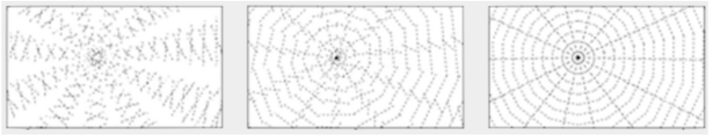

No, not at all ⊢——————————⊣ Yes, very much

Item 5: Did you see any tunnels, geometric forms organised around a distant focal point?

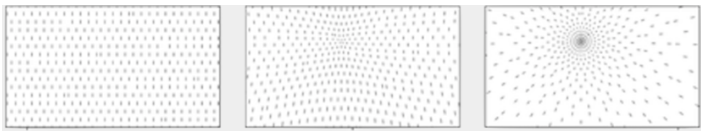

No, not at all ⊢——————————⊣ Yes, very much

Item 6: Did you see any spirals?

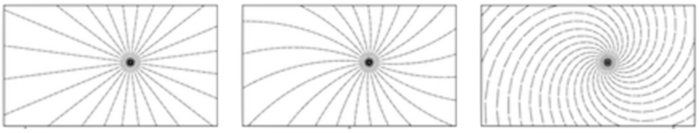

No, not at all ⊢——————————⊣ Yes, very much

Item 7: Did your visual experience consist of geometric formations including points, lines, shapes and/or patterns?

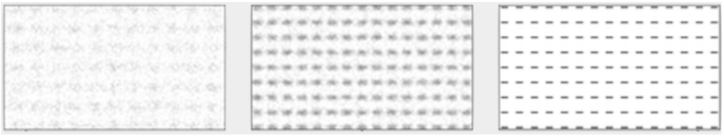

No, not at all — Yes, very much

Item 8: Did you see any shapes rippling outwards from a focal point?

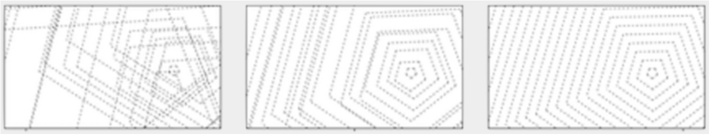

No, not at all — Yes, very much

Item 9: Did you see any scattered geometric forms?

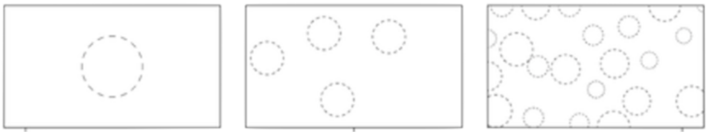

No, not at all — Yes, very much

Item 10: Did you see any flowing lines like fur or hairs?

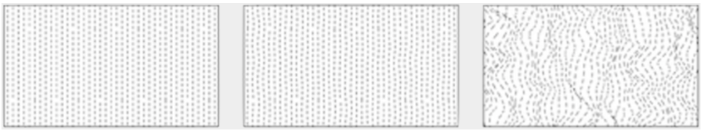

No, not at all — Yes, very much

Item 11: Did you see any TV snow, TV static or "salt and pepper" texture?

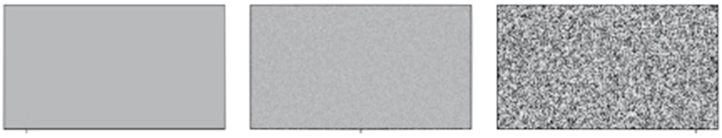

No, not at all — Yes, very much

Item 12: Did you see any blobs of light or colour?

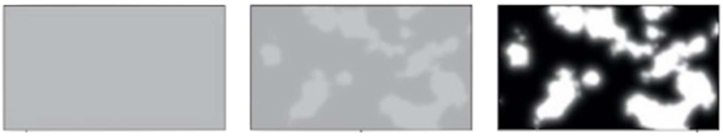

No, not at all — Yes, very much

Item 13: Did your visual experience contain a high level of detail (fine-grained)?

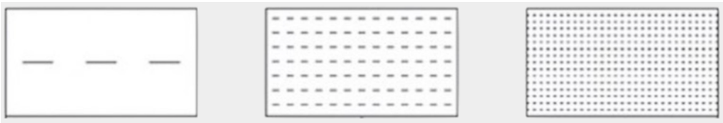

No, not at all — Yes, very much

Item 14: Did your visual experience continuously change or evolve over time?

No, not at all ⊢——⊣ Yes, very much

Item 15: Did your visual experience include motion? Such as patterns or shapes that moved across, around or within your visual field?

No, not at all ⊢——⊣ Yes, very much

Item 16: Did your visual experience contain a high level of randomness or chaos?

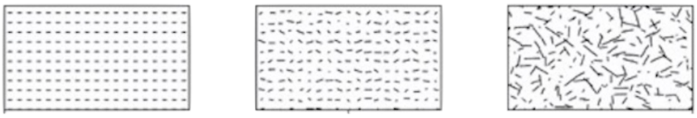

No, not at all ⊢——⊣ Yes, very much

Item 17: Did you see any paisley patterns?

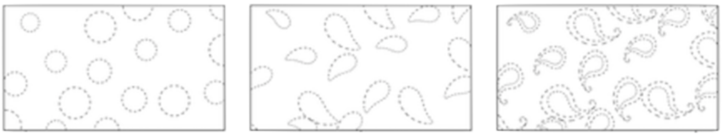

No, not at all ⊢——⊣ Yes, very much

Item 18: Did your visual experience consist of things such as objects, people or places similar to what you might see with your eyes open?

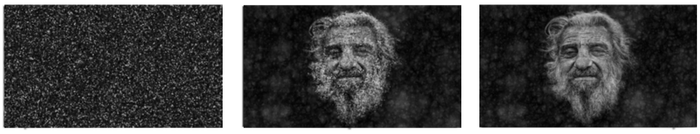 *

No, not at all ⊢——⊣ Yes, very much

Item 19: Was your visual experience dominated by only uniform light or colour and nothing else?

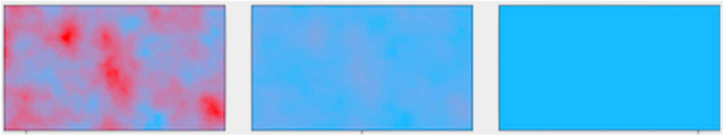

No, not at all ⊢——⊣ Yes, very much

Item 20: Did your visual experience consist of anything in addition to blackness and/or flashes?

No, not at all ⊢——⊣ Yes, very much

* This image is visually similar but not identical to the original image used in study implementation, therefore it is placed here for illustrative purposes. Note that all future versions of the SVES will use the image provided here, which complies with the CC BY 4.0 license.
