# Supplementary material for: Effect of frequency and rhythmicity on flicker light-induced hallucinatory phenomena
Source: PLoS One. 2023 Apr 11;18(4):e0284271. doi: 10.1371/journal.pone.0284271 (PMC10089352; doi:10.1371/journal.pone.0284271)
Supplement: Attachment — Submitted filename: Response to Reviewers.docx [file pone.0284271.s003.docx]

PONE-D-22-33362

Effect of frequency and rhythmicity on flicker light-induced hallucinatory phenomena

PLOS ONE

Dear Dr. Schmidt,

Thank you for submitting your manuscript to PLOS ONE. After careful consideration, we feel that it has merit but does not fully meet PLOS ONE’s publication criteria as it currently stands. Therefore, we invite you to submit a revised version of the manuscript that addresses the points raised during the review process.

==============================
Please address all reviewers' comments.
==============================

Please submit your revised manuscript by Mar 12 2023 11:59PM. If you will need more time than this to complete your revisions, please reply to this message or contact the journal office at [plosone@plos.org](mailto:plosone@plos.org). When you're ready to submit your revision, log on to <https://www.editorialmanager.com/pone/> and select the 'Submissions Needing Revision' folder to locate your manuscript file.

Please include the following items when submitting your revised manuscript:

- A rebuttal letter that responds to each point raised by the academic editor and reviewer(s). You should upload this letter as a separate file labeled 'Response to Reviewers'.
- A marked-up copy of your manuscript that highlights changes made to the original version. You should upload this as a separate file labeled 'Revised Manuscript with Track Changes'.
- An unmarked version of your revised paper without tracked changes. You should upload this as a separate file labeled 'Manuscript'.

If you would like to make changes to your financial disclosure, please include your updated statement in your cover letter. Guidelines for resubmitting your figure files are available below the reviewer comments at the end of this letter.

If applicable, we recommend that you deposit your laboratory protocols in protocols.io to enhance the reproducibility of your results. Protocols.io assigns your protocol its own identifier (DOI) so that it can be cited independently in the future. For instructions see: <https://journals.plos.org/plosone/s/submission-guidelines#loc-laboratory-protocols>. Additionally, PLOS ONE offers an option for publishing peer-reviewed Lab Protocol articles, which describe protocols hosted on protocols.io. Read more information on sharing protocols at <https://plos.org/protocols?utm_medium=editorial-email&utm_source=authorletters&utm_campaign=protocols>.

We look forward to receiving your revised manuscript.

Kind regards,

Manuel Spitschan
Academic Editor
PLOS ONE

Journal Requirements:

When submitting your revision, we need you to address these additional requirements.

1. Please ensure that your manuscript meets PLOS ONE's style requirements, including those for file naming. The PLOS ONE style templates can be found at 
<https://journals.plos.org/plosone/s/file?id=wjVg/PLOSOne_formatting_sample_main_body.pdf> and <https://journals.plos.org/plosone/s/file?id=ba62/PLOSOne_formatting_sample_title_authors_affiliations.pdf>

Authors’ response:

Thank you for highlighting these style requirements to us! We have included an asterix to show the corresponding author and included Dr. Schmidt’s email address on the title page. We also removed postal codes from the affiliations and started the line numbering from the abstract onwards. We changed the heading and subheading formatting as well as figure citations.

2. We note that the grant information you provided in the ‘Funding Information’ and ‘Financial Disclosure’ sections do not match.

When you resubmit, please ensure that you provide the correct grant numbers for the awards you received for your study in the ‘Funding Information’ section.

Authors’ response:

The obtained funding does not have a grant number. Therefore, we provided a description of the funding.

3. Thank you for stating in your Funding Statement: 
"The investigator-initiated study was financially supported by a donation from Lumenate Growth Ltd to TTS."

Please provide an amended statement that declares *all* the funding or sources of support (whether external or internal to your organization) received during this study, as detailed online in our guide for authors at <http://journals.plos.org/plosone/s/submit-now>.  Please also include the statement “There was no additional external funding received for this study.” in your updated Funding Statement. 
Please include your amended Funding Statement within your cover letter. We will change the online submission form on your behalf.

Authors’ response:

Thank you for highlighting this to us. We would like to change our Funding Statement to:

The investigator-initiated study was financially supported by a donation from Lumenate Growth Ltd to Freie Universität Berlin allocated to TTS. There was no additional external funding received for this study.

4. Thank you for stating the following in the Competing Interests section:
"I have read the journal's policy and the authors of this manuscript have
the following competing interests:
TTS: This research was supported by a unrestricted donation from Lumenate
Growth ltd to Freie Universität Berlin allocated to TTS."

We note that you received funding from a commercial source: Lumenate Growth ltd 
Please provide an amended Competing Interests Statement that explicitly states this commercial funder, along with any other relevant declarations relating to employment, consultancy, patents, products in development, marketed products, etc.

Within this Competing Interests Statement, please confirm that this does not alter your adherence to all PLOS ONE policies on sharing data and materials by including the following statement: "This does not alter our adherence to PLOS ONE policies on sharing data and materials.” (as detailed online in our guide for authors <http://journals.plos.org/plosone/s/competing-interests>).  If there are restrictions on sharing of data and/or materials, please state these. Please note that we cannot proceed with consideration of your article until this information has been declared.

Please include your amended Competing Interests Statement within your cover letter. We will change the online submission form on your behalf.

Authors’ response:

We thank you for bringing this to our attention. We would like to amend our Competing Interests statement to:

I have read the journal's policy and the authors of this manuscript have the following competing interests:

TTS: This research was supported by an unrestricted donation from Lumenate

Growth ltd to Freie Universität Berlin allocated to TTS. This does not alter our adherence to PLOS ONE policies on sharing data and materials. The funder had no role in study design, data collection and analysis, decision to publish, or preparation of the manuscript.

5. We note that Figures 1 and S1 Appendix in your submission contain copyrighted images. All PLOS content is published under the Creative Commons Attribution License (CC BY 4.0), which means that the manuscript, images, and Supporting Information files will be freely available online, and any third party is permitted to access, download, copy, distribute, and use these materials in any way, even commercially, with proper attribution. For more information, see our copyright guidelines: <http://journals.plos.org/plosone/s/licenses-and-copyright>.
 
We require you to either (1) present written permission from the copyright holder to publish these figures specifically under the CC BY 4.0 license, or (2) remove the figures from your submission:
 
a. You may seek permission from the original copyright holder of Figures 1 and S1 Appendix to publish the content specifically under the CC BY 4.0 license. 
 
We recommend that you contact the original copyright holder with the Content Permission Form (<http://journals.plos.org/plosone/s/file?id=7c09/content-permission-form.pdf>) and the following text:
“I request permission for the open-access journal PLOS ONE to publish XXX under the Creative Commons Attribution License (CCAL) CC BY 4.0 (<http://creativecommons.org/licenses/by/4.0/>). Please be aware that this license allows unrestricted use and distribution, even commercially, by third parties. Please reply and provide explicit written permission to publish XXX under a CC BY license and complete the attached form.”
 
Please upload the completed Content Permission Form or other proof of granted permissions as an ""Other"" file with your submission. 
 
In the figure caption of the copyrighted figure, please include the following text: “Reprinted from [ref] under a CC BY license, with permission from [name of publisher], original copyright [original copyright year].”
 
b. If you are unable to obtain permission from the original copyright holder to publish these figures under the CC BY 4.0 license or if the copyright holder’s requirements are incompatible with the CC BY 4.0 license, please either i) remove the figure or ii) supply a replacement figure that complies with the CC BY 4.0 license. Please check copyright information on all replacement figures and update the figure caption with source information. If applicable, please specify in the figure caption text when a figure is similar but not identical to the original image and is therefore for illustrative purposes only.

6. We note that Figure S1 Appendix includes an image of a participant in the study. 
As per the PLOS ONE policy (<http://journals.plos.org/plosone/s/submission-guidelines#loc-human-subjects-research>) on papers that include identifying, or potentially identifying, information, the individual(s) or parent(s)/guardian(s) must be informed of the terms of the PLOS open-access (CC-BY) license and provide specific permission for publication of these details under the terms of this license. Please download the Consent Form for Publication in a PLOS Journal (<http://journals.plos.org/plosone/s/file?id=8ce6/plos-consent-form-english.pdf>). The signed consent form should not be submitted with the manuscript, but should be securely filed in the individual's case notes. Please amend the methods section and ethics statement of the manuscript to explicitly state that the patient/participant has provided consent for publication: “The individual in this manuscript has given written informed consent (as outlined in PLOS consent form) to publish these case details”.

If you are unable to obtain consent from the subject of the photograph, you will need to remove the figure and any other textual identifying information or case descriptions for this individual.

Authors’ response:

With regards to Figure 1, this cannot be true as we designed all figure elements by ourselves. We suspect that an algorithm may found a similarity of the sitting stick figure that we have included in the figure. This figure element was created by ourselves and therefore cannot be under copyright.

Regarding the image in S1 Appendix, while we note that the image is not of a participant in the study, we cannot obtain permission from the original copyright holder to publish this image. Therefore, we use a replacement image that complies with the CC by 4.0 license and is suitable for PLOS One publication. The replacement image is here: <https://www.pexels.com/photo/gray-scale-photo-of-male-with-beard-818261/> and the corresponding license information is here: https://www.pexels.com/license/

7. Please review your reference list to ensure that it is complete and correct. If you have cited papers that have been retracted, please include the rationale for doing so in the manuscript text, or remove these references and replace them with relevant current references. Any changes to the reference list should be mentioned in the rebuttal letter that accompanies your revised manuscript. If you need to cite a retracted article, indicate the article’s retracted status in the References list and also include a citation and full reference for the retraction notice.

Authors’ response:

Thank you for highlighting this. We corrected the formatting of references 12 (lines 655-6), 31 (702-3) and 62 (789-91). We did not identify that any of the articles cited in this manuscript have since been retracted.

[Note: HTML markup is below. Please do not edit.]

Reviewers' comments:

Reviewer's Responses to Questions

**Comments to the Author**

1. Is the manuscript technically sound, and do the data support the conclusions?

The manuscript must describe a technically sound piece of scientific research with data that supports the conclusions. Experiments must have been conducted rigorously, with appropriate controls, replication, and sample sizes. The conclusions must be drawn appropriately based on the data presented.

Reviewer #1: Yes

Reviewer #2: Yes

2. Has the statistical analysis been performed appropriately and rigorously?

Reviewer #1: Yes

Reviewer #2: Yes

3. Have the authors made all data underlying the findings in their manuscript fully available?

The [PLOS Data policy](http://www.plosone.org/static/policies.action#sharing) requires authors to make all data underlying the findings described in their manuscript fully available without restriction, with rare exception (please refer to the Data Availability Statement in the manuscript PDF file). The data should be provided as part of the manuscript or its supporting information, or deposited to a public repository. For example, in addition to summary statistics, the data points behind means, medians and variance measures should be available. If there are restrictions on publicly sharing data—e.g. participant privacy or use of data from a third party—those must be specified.

Reviewer #1: Yes

Reviewer #2: Yes

4. Is the manuscript presented in an intelligible fashion and written in standard English?

PLOS ONE does not copyedit accepted manuscripts, so the language in submitted articles must be clear, correct, and unambiguous. Any typographical or grammatical errors should be corrected at revision, so please note any specific errors here.

Reviewer #1: Yes

Reviewer #2: Yes

5. Review Comments to the Author

Please use the space provided to explain your answers to the questions above. You may also include additional comments for the author, including concerns about dual publication, research ethics, or publication ethics. (Please upload your review as an attachment if it exceeds 20,000 characters)

**Reviewer #1**:

Review of “Effect of frequency and rhythmicity on flicker light-induced hallucinatory phenomena”.

The authors report an experiment investigating the frequency and rhythmicity of flickering light on simple visual hallucinations. They found the experience of visual hallucinations to be maximal at 10 Hz and that rhythmic light resulted in stronger hallucinations than an arrhythmic control condition.

Overall I find no major flaws in the study. This is a very interesting topic and an important step towards understanding the phenomena.

The following should be considered minor revisions/suggestions.

Authors’ response:

We are very grateful for the positive feedback towards our study. Thank you!

Some additional information on the experimental setup should be added to the methods section. There were 12 LEDs, presumably these were white LEDs? This should be stated for clarity.
What pattern were the LEDs positioned in, e.g. in a circle, a grid? How far apart?
Importantly, the visual angle of the light source should be reported because peripheral flickering light is perceived differently from flickering light on the fovea. Although the light is diffuse because the eyes are closed, the visual angle of the light source could be a factor on the resulting perceptions. This can simply be calculated from the distance and width of the light source.

Authors’ response:

Thank you for highlighting the need for a more detailed description. We have included the following additional information in the methods section (page 6; lines 121-26):

“A custom stroboscope was constructed by Lumenate Growth Inc. (Bristol, United Kingdom) to generate light stimulation. It consists of twelve 4500k J2 6V white LEDs organised in a three-by-four grid with dimensions 128 x 176mm (width x height). The lamp was set to deliver 5,520 Lumens over participants’ eyes (maximum capacity is 10,360 Lumens). Positioned approximately 150cm from participants, the LEDs were within a visual angle of approximately 6.5°, while the setup assured that illumination of the visual field was experienced as homogenous.”

A potential factor which should be considered is the position of the eyes when eyelids are closed. In my experience participants sometimes report moving their eyes to look away from a bright light sources with eyes closed, as this reduces the unpleasantness of bright light. Furthermore, it is known that it is difficult to maintain a consistent position of the eye with eyes closed, so the position might change over time. This is not a critical problem for the current study because there is no reason to suspect that the eye position was different across control conditions, but it should be mentioned in the discussion as a potential source of variability in the subjective experience.

Authors’ response:

We thank the reviewer for this point! We agree that it is important to consider different possibilities that would affect the subjective experience. We included this as a point of discussion on page 21 (lines 455-7):

“Future studies could incorporate eye tracking sensors that monitor participant eye movements during the flicker experience to explore whether participants’ eye position and movement adds to variability in the subjective experience.”

A second control condition using paired arrhythmic stimulation was used. It is not clear why an additional control condition is needed, when the arrhythmic condition controls for rhythmicity. Perhaps the authors could explain the decision to add an extra control condition in more detail in the introduction. For example, do the authors hypothesize that the decreases in occipitotemporal EEG activity found by Ffytche is not present in the normal arrhythmic control condition? The authors report that the paired arrhythmic stimulation can be considered more arrhythmic, it would be good if they could elaborate on why this is the case. Is it simply a greater variation in inter flash intervals? Do the authors have any hypothesis as to why the reduction in visual effects was most pronounced for the arrhythmic pairs condition?

Authors’ response:

We thank the reviewer for pointing out that the reasoning behind the control conditions needs more clarity. Following the reviewer’s advice, we now report a measure of the degree of arrhythmicity of the two arrhythmic conditions, which were designed with the intention that the more irregular the stimulation is, the less subjective effects we expected. We used the root-mean-square of successive differences (in time) between flashes. This is a measure commonly used to quantify heart rate variability (RMSSD; Shaffer and Ginsberg, 2017). The paired control condition has a RMSSD value more than two times higher than the normal control at the same frequency level. Please note: The RMSSD value decreased with increasing frequency.

In the introduction (lines 94-6): “We utilise two arrhythmic conditions that vary in their degree of arrhythmicity. We hypothesise that higher variability in inter-flash intervals (i.e., greater arrhythmicity) will lead to a greater reduction in subjective effects.”

In the methods section (lines 143-7): “The Arrhythmic_pairs_ condition contains IFIs with higher variability than the Arrhythmic_norm_ at each frequency level (excluding the constant inter-pair interval). This was determined using the root mean squared of successive differences (RMSSD), which is commonly used for calculating heart rate variability [29] (e.g., at 10Hz, Arrhythmic_pairs_: RMSSD = 166.57, Arrhythmic_norm_: 64.22).”

In the discussion section (lines 551-4): “In our study, we found the greatest differences in reported hallucinatory phenomena between Rhythmic and the Arrhythmic_pairs_ control, which has more arrhythmicity than the normally distributed control, as determined by the RMSSD of IFIs (see Methods).”

The authors discuss the possibility of neural entrainment being responsible for the generation of simple visual hallucinations. While I agree that neural entrainment is a possible (even likely) explanation, there is a debate in the EEG literature as to whether the frequency specific neural responses to visual flicker (SSVEPs) are the result of entrainment of ongoing oscillations, or a resonance phenomena where the cortex has a preferred frequency of stimulation. For example, Capilla et. al. (2011) argued SSVEPs can be explained as a superposition of transient responses to the visual stimulation. In a recent study we have shown that different cortical sources of alpha oscillations show very different steady state responses to visual flicker, which vary depending on the distance of the flicker frequency and the individual’s alpha frequency (Nuttall et. al. 2022). In the current study the authors have shown that rhythmicity is important for the hallucinatory visual effect, so an interaction between neural oscillations and the flicker is likely, but entrainment is only one possible explanation. Distinguishing between entrainment and resonance is difficult and the exact definition of entrainment is not always clearly defined, see Haegens (2020) for a good discussion. It is quite possible that different brain areas respond to rhythmic stimulation in a variety of ways, with some showing entrainment of an ongoing oscillation, and some simply responding preferentially to an optimal frequency of rhythmic input. I appreciate that a full discussion of these issues is beyond the scope of the current article, but it would be good to acknowledge the possibility of different neural mechanisms which might explain this effect.

James Dowsett

References:

Capilla A, Pazo-Alvarez P, Darriba A, Campo P, Gross J (2011) Steady-State Visual Evoked Potentials Can Be Explained by Temporal Superposition of Transient Event-Related Responses. PLoS ONE 6(1): e14543.

Nuttall, R., Jäger, C., Zimmermann, J., Archila-Melendez, M. E., Preibisch, C., Taylor, P., ... & Dowsett, J. (2022). Evoked responses to rhythmic visual stimulation vary across sources of intrinsic alpha activity in humans. Scientific reports

Saskia Haegens (2020): Entrainment revisited: a commentary on Meyer, Sun,
and Martin (2020), Language, Cognition and Neuroscience, DOI: 10.1080/23273798.2020.1758335

Authors’ response:

Thank you, James Dowsett, for this very helpful advice. We also thank you for providing these references. We totally agree that it is a bit too simplistic to just say that it might be entrainment which drives the subjective experiences. We have now added the following on page 24 (lines 545-7):

“Haegens [44] defined entrainment as the phase alignment of existing brain oscillations to an external periodic stimulus, which continues for several cycles after stimulus termination.”

And page 24-25 (lines 557-69):

“In this light, it should be considered that other mechanisms can also contribute to oscillatory activity, such as the superposition of event-related responses, which are evoked cortical responses to visual stimulation that add onto, but do not interact with, ongoing oscillations [48]. Moreover, a recent study using rhythmic flickering checkerboards found evidence for both frequency-specific neural responses, supporting the entrainment model, and frequency-independent resonance phenomena, supporting the superposition model [49]. As we found that intensity of FLS effects was affected by frequency, it is likely that underlying frequency-specific neural responses, such as entrainment, contribute to FLS effects. However, future EEG research is necessary to test whether FLS-induced neural responses satisfy the criteria for entrainment [44] and if there is markedly less entrainment elicited by arrhythmic conditions. In doing so, evidence can be provided to determine whether neural entrainment mediates the effect of frequency and rhythmicity on the flicker-induced subjective experience.”

**Reviewer #2:**

This paper examines – as summarised in the title – the effect of frequency and rhythmicity on various flicker-induced visual hallucinations. As described in the paper, 10-Hz visual stimulation has been reported to lead to strongest hallucinations (as compared to other stimulation rates), but the effect of rhythmicity remained unclear due to the lack of well-designed arrhythmic control conditions. Such conditions, along with novel questionnaires, were used in the current study. The authors were able to confirm the advantage of 10-Hz flicker (among several other rates) to induce visual hallucinations. In addition, they show that, in particular for 10 Hz, rhythmic stimulation leads to stronger hallucinatory effects than arrhythmic control stimulation.
This paper is very well written and the rationale is easy to follow. I cannot see any major issues with this work and believe that this is a well-designed and executed study. I do have two minor comments:

Authors’ response:

We would like to warmly thank the reviewer for the positive feedback towards our study.

- I was a bit confused about arrhythmic pairs control condition. The rate (frequency) of paired flashes always seems faster than in the corresponding rhythmic condition. Given the effect of rate on perception, I wonder if this can lead to an apparent effect of rhythmicity that is due to this difference in “local” frequency/rate.

Authors’ response:

Thank you for this point. Indeed, it is an important point to note that local frequencies can be higher when inter-flash intervals (IFIs) are randomized. We have randomized the IFIs within one second. Thereby, we limited the possibility for longer phases of such local higher frequencies as they will be automatically interrupted by a longer IFI. Nevertheless, it is a possibility that these higher frequencies contribute to the subjective experiences. As we found that the extent of subjective experiences peak at 10Hz, an increase in frequency appears to not linearly relate to the subjective experiences, we consider it however unlikely. To clearly emphasize the possibility of such an effect we have added the following to the manuscript on page 22 (lines 469-74):

“In case of higher frequencies (i.e., 18Hz), flicker trains of higher frequency due to IFI randomization were more likely to occur than in the other conditions, with local frequency of up to 37 Hz (compare Fig 1). Due to the randomization of IFIs within one second, these trains were very short and interrupted by longer IFIs, making it unlikely that they were majorly driving the subjective experiences.”

- For some statistical tests, the authors report a reliable interaction of rhythmicity and frequency. In some cases (e.g., page 13), it remains unclear which combination of conditions has produced the interaction. This could be described in more detail, in particular as main effects can be difficult to interpret in the presence of such an interaction.

Authors’ response:

We thank the reviewer for drawing our attention to this issue. While the supplementary information (S2 Tables) gives a full report of the different interaction effects, we notice that the highlighted findings in the main text should be improved. Therefore, we decided to include Tukey test reports stating the directionality of significant interactions for SVES simple hallucination ratings (lines 284-91) and visual dynamics (lines 318-23).

“For interaction effects, post-hoc Tukey tests showed that the highest increase in reports of simple visual hallucination was between Rhythmic and Arrhythmic_pairs_ stimulation at 10Hz (p < .001) and remained significant for 18Hz (p = .003) and 8Hz (p = .01) [See S2 Tables for full report of Tukey tests]. Furthermore, during Rhythmic stimulation, reports of simple visual hallucinations are significantly higher during 8Hz, 10Hz and 18Hz compared to 3Hz (all p < .001) [See S2 Tables for full report of Tukey tests]. Fig 2A summarises the interaction between rhythmicity and frequency on ratings of simple visual hallucinations.”

“For interaction effects, post-hoc Tukey tests revealed that, during Rhythmic stimulation, 8 Hz, 10 Hz and 18 Hz elicited higher ratings of visual dynamics than 3 Hz (p < .001). Moreover, ratings were higher for Rhythmic compared to Arrhythmic_pairs_ at 8 Hz, 10 Hz and 18 Hz (all p < 0.001) [See S2 Tables for Tukey test results]. At 10Hz, visual dynamics were also higher for Rhythmic compared to Arrhythmic_norm_ (p = .03) [See S2 Tables for full report of Tukey tests].”

6. PLOS authors have the option to publish the peer review history of their article ([what does this mean?](https://journals.plos.org/plosone/s/editorial-and-peer-review-process#loc-peer-review-history)). If published, this will include your full peer review and any attached files.


If you choose “no”, your identity will remain anonymous but your review may still be made public.


**Do you want your identity to be public for this peer review?** For information about this choice, including consent withdrawal, please see our [Privacy Policy](https://www.plos.org/privacy-policy).

Reviewer #1: **Yes:** James Dowsett

Reviewer #2: **Yes:** Benedikt Zoefel

[NOTE: If reviewer comments were submitted as an attachment file, they will be attached to this email and accessible via the submission site. Please log into your account, locate the manuscript record, and check for the action link "View Attachments". If this link does not appear, there are no attachment files.]

While revising your submission, please upload your figure files to the Preflight Analysis and Conversion Engine (PACE) digital diagnostic tool, <https://pacev2.apexcovantage.com/>. PACE helps ensure that figures meet PLOS requirements. To use PACE, you must first register as a user. Registration is free. Then, login and navigate to the UPLOAD tab, where you will find detailed instructions on how to use the tool. If you encounter any issues or have any questions when using PACE, please email PLOS at [figures@plos.org](mailto:figures@plos.org). Please note that Supporting Information files do not need this step.

Additional References

Grüsser, OJ., Hagner, M. On the history of deformation phosphenes and the idea of internal light generated in the eye for the purpose of vision. Doc Ophthalmol 74, 57–85 (1990). https://doi.org/10.1007/BF00165665

Shaffer F, Ginsberg JP. An Overview of Heart Rate Variability Metrics and Norms. Front Public Health. 2017 Sep 28;5:258. doi: 10.3389/fpubh.2017.00258. PMID: 29034226; PMCID: PMC5624990.
